# Clinical Predictors and Determinants of Mpox Complications in Hospitalized Patients: A Prospective Cohort Study from Burundi

**DOI:** 10.3390/v17040480

**Published:** 2025-03-27

**Authors:** Liliane Nkengurutse, John O. Otshudiema, Godefroid Kamwenubusa, Issa Diallo, Odette Nsavyimana, Jean Claude Mbonicura, Jean Claude Nkurunziza, Fidèle Cishahayo, Dieudonné Niyongere, Bonite Havyarimana, Déo Simbarariye, Marc Nimburanira, Bosco Ntiranyibagira, Senya Diane Nzeyimana, Brigitte Ndelema, Denise Nkezimana, Parfait Shingiro, Aimable Sibomana, Stany Nduwimana, Freddy Nyabenda, Alexis Niyomwungere, Mamadou Zongo, Abdoulaye Bousso, Samuel Boland, Jeanine Ndayisenga, Dionis Nizigiyimana, Joseph Nyandwi, Alimuddin Zumla, Rosamund F. Lewis, Stanislas Harakandi

**Affiliations:** 1 Centre des Opérations d’Urgence de Santé Publique (COUSP/PHEOC Burundi), Rohero I, Bujumbura Mairie, Bujumbura J978+9V4, Burundi; lilianenkengurutse@gmail.com (L.N.); kamwenubusa.godefroid@yahoo.com (G.K.); parfaitshingiro@gmail.com (P.S.); aimablesibo22@gmail.com (A.S.); nyabendafredy@gmail.com (F.N.); 2 World Health Organization, WHO Burundi, 4 Avenue Muramwya, Rohero I, Bujumbura Mairie, Bujumbura J978+9V4, Burundi; diallois@who.int (I.D.); nzeyimanas@who.int (S.D.N.); ndelemab@who.int (B.N.); nkezimanad@who.int (D.N.); nduwimanas@who.int (S.N.); niyomwungerea@who.int (A.N.); zongom@who.int (M.Z.); abousso@who.int (A.B.); 3 African Region Mpox Incident Management Support Team, World Health Organization Regional Office for Africa, Cité du Djoué OMS, Brazzaville P.O. Box 06, Congo; sboland@who.int; 4 Mpox Treatment Center, Centre Hospitalo-Universitaire de Kamenge (CHUK), Rohero I, Bujumbura Mairie, Bujumbura J978+9V4, Burundi; odettensa2019@gmail.com (O.N.); jean-claudembonicura@ubu.edu.bi (J.C.M.); jean-claude.nkurunziza@ubu.edu.bi (J.C.N.); harakandis@yahoo.fr (S.H.); 5 Mpox Treatment Center, Clinique Prince Louis Rwagasore (CPLR), Rohero I, Bujumbura Mairie, Bujumbura J978+9V4, Burundi; fidelec84@gmail.com (F.C.); nidodo1@gmail.com (D.N.); bonitehavyarimana@gmail.com (B.H.); 6 Mpox Treatment Center, Hôpital Militaire de Kamenge (HMK), Rohero I, Bujumbura Mairie, Bujumbura J978+9V4, Burundi; sidouglas2009@yahoo.fr (D.S.); directionhmk@gmail.com (M.N.); boscontiranyibagira@gmail.com (B.N.); 7 Institut National de Santé Publique (INSP), Rohero I, Bujumbura Mairie, Bujumbura J978+9V4, Burundi; ndayisengajeanine38@gmail.com (J.N.); nizigiyimana.dionis@gmail.com (D.N.); nyandwijo@yahoo.fr (J.N.); 8 Division of Infection and Immunity, Centre for Clinical Microbiology, University College London, London NW1 2PG, UK; a.zumla@ucl.ac.uk; 9 National Institutes of Healthcare Research (NIHR), Biomedical Research Centre, UCL Hospitals NHS Foundation Trust, London NW1 2PG, UK; 10 Health Emergencies Programme, World Health Organization, Avenue Appia 20, 1211 Geneva, Switzerland; lewisr@who.int

**Keywords:** Burundi, mpox (monkeypox), prospective studies, resource-limited settings, conjunctivitis, risk assessment

## Abstract

(1) Objectives: Studies on mpox patterns, severity predictors, and public health impacts in Burundi remain limited. Therefore, we aimed to identify the clinical predictors and determinants of mpox complications among hospitalized patients in Bujumbura, Burundi, during an active outbreak. (2) Methods: We conducted a prospective cohort study of laboratory-confirmed mpox cases across three treatment centers (July–October 2024). Clinical characteristics and outcomes were assessed through a systematic review of medical and laboratory records supplemented by structured interviews with patients or caregivers. Risk factors for disease complications were evaluated using multivariate Firth penalized logistic regression. (3) Results: Complications developed in 3.1% of 850 patients (54.4% male; median age, 20.3 years). Conjunctivitis (odds ratio [OR]: 27.30; 95% confidence interval [CI], 7.67–122.23) and sore throat (OR: 12.63; 95% CI, 5.78–30.21) were significant predictors of severe disease progression. Conversely, generalized rash (OR, 0.10; 95% CI, 0.04–0.24) and lymphadenopathy (OR, 0.24; 95% CI, 0.08–0.62) were associated with a mild disease course. Sexual transmission was the predominant route of infection (58.6%). (4) Conclusions: Noncutaneous manifestations, particularly conjunctivitis and sore throat, are early indicators of mpox severity. These findings inform clinical risk stratification in resource-limited settings and highlight the need for further investigation of pathophysiological mechanisms.

## 1. Introduction

Mpox (formerly monkeypox) is caused by the monkeypox virus (MPXV), an enveloped double-stranded DNA virus belonging to the Orthopoxvirus genus in the Poxviridae family. There are two distinct clades of the virus: clade I (with subclades Ia and Ib) and clade II (with subclades IIa and IIb) [1]. While MPXV is closely related to variola virus (the causative agent of smallpox), these viruses exhibit distinct clinical and epidemiological characteristics [2]. The disease typically begins with a prodromal phase marked by fever, headache, and distinctive lymphadenopathy, which differentiates it from smallpox [2]. Both diseases produce pustular lesions, though mpox lesions characteristically appear more concentrated on the extremities and face. Mpox generally presents as a milder disease with lower mortality rates compared to historical smallpox [2]. Initially documented through sporadic outbreaks in Central and West Africa [1], mpox transmission occurs through both zoonotic and human-to-human routes, primarily via close contact and fomites [1,2]. The impact has been particularly severe in resource-limited settings in Africa, where healthcare systems continue to face challenges in outbreak management and surveillance, as demonstrated by the 2017 Nigerian outbreak experience [3].

Following the first Public Health Emergency of International Concern declaration for mpox in 2022 and its termination in May 2023, the emergence of a new MPXV subclade (clade Ib) in the Democratic Republic of the Congo (DRC) in September 2023 led to the second Public Health Emergency of International Concern declaration by the World Health Organization (WHO) in August 2024. Concurrently, the Africa Centres for Disease Control and Prevention issued its first declaration of a Public Health Emergency of Continental Security as the outbreak had spread to multiple neighboring countries, including Burundi [4].

Burundi reported its initial mpox case on 25 July 2024, with the city of Bujumbura becoming the national epicenter, accounting for 59% of the confirmed cases by 26 October 2024 [Unpublished Burundi mpox situation report # 093, Ministry of Health. 26 October 2024]. While substantial research has been conducted in low-resource settings, such as Nigeria and the DRC [5,6], understanding of mpox patterns, severity predictors, and public health impact remains limited in Burundi, not having been reported before 2024. The current literature describes common manifestations, including fever, lymphadenopathy, and pustular rash [7,8]; however, the prognostic significance of noncutaneous symptoms remains poorly understood.

This study aimed to identify the clinical predictors and determinants of mpox complications among hospitalized patients in Bujumbura, Burundi, to enhance early detection and clinical management in resource-limited settings during an active outbreak.

## 2. Patients and Methods

### 2.1. Study Design and Settings

This prospective cohort study was conducted at three primary treatment centers in Bujumbura, Burundi: the Centre Hospitalo-Universitaire de Kamenge, Clinic Prince Louis Rwagasore, and Hôpital Militaire de Kamenge. This study was conducted between 25 July 2024, and 26 October 2024, coinciding with the initial peak of the ongoing mpox outbreak in Burundi.

### 2.2. Participant Selection and Case Definitions

All 850 patients with laboratory-confirmed mpox who were hospitalized at the three selected mpox treatment centers in Bujumbura during the defined period were included, with no exclusion criteria. No exclusion criteria were applied to maintain study representativeness. Standardized case definitions based on WHO criteria were adopted by the Burundi Ministry of Health and implemented across all participating centers. A suspected case was defined as an individual presenting with an unexplained acute skin rash, mucosal lesions, or lymphadenopathy, accompanied by fever (>38.5 °C) and associated symptoms, following the exclusion of measles, varicella zoster, and other skin lesion-related diseases. A probable case was defined as a suspected case presenting with skin lesions and an established epidemiological link (contact with a confirmed or probable case within 21 days of symptom onset) without laboratory confirmation. A confirmed case was defined as a suspected case with positive laboratory results through a generic real-time reverse transcription-polymerase chain reaction. Regular monthly audits were conducted to ensure consistent application.

In Burundi, all laboratory-confirmed mpox cases required mandatory hospitalization, regardless of disease severity. With the provision of free health care services, all patients completed their hospital stay and underwent a standardized 28-day post-discharge follow-up. Of the 1441 laboratory-confirmed mpox cases in Burundi, 850 (59%) from Bujumbura’s three treatment centers were enrolled in this study (Burundi Ministry of Health, unpublished situation report #093, 2024).

Disease severity was evaluated using two standardized approaches: (1) a modified Mpox Severity Scoring System [9] that incorporates skin lesions, mucosal involvement, and systemic symptoms and (2) WHO clinical criteria, which categorized cases as mild (≤5 skin lesions), moderate (6–100 lesions or mild complications), or severe (>100 lesions and/or serious complications, such as secondary bacterial infections, encephalitis, pneumonia, genital necrosis, or ocular involvement). Conjunctivitis was classified as a mild condition, whereas keratitis and sight-threatening conditions were categorized as severe ocular complications. Lymphadenopathy, a characteristic feature of mpox, was analyzed as a presenting clinical feature rather than as a severity indicator or complication.

### 2.3. Data Collection

For mpox laboratory confirmation, we performed both generic real-time and clade Ib-specific reverse transcription-polymerase chain reaction amplification using the TaqMan Fast Advanced Master Mix (Thermo Fisher Scientific, Waltham, MA, USA). We used various TaqMan-based assays for MPXV detection. This mpox confirmation was performed at the National Reference Public Health Laboratory of the National Institute of Public Health (Institut National de Santé Publique) [10,11].

We used a mixed-methods approach combining structured interviews with patients or their parents/caregivers performed by clinicians and surveillance officers, data from electronic health records at “Open Clinics”, national surveillance databases, case investigations, contact tracing, and records of laboratory polymerase chain reactions. Physicians used a WHO-adapted structured questionnaire to collect confidential patient information, including demographics, symptoms, complications, treatments, and outcomes. Clinicians’ training included human immunodeficiency virus (HIV) screening according to Burundi’s guidelines (unpublished), which required informed consent. The center protocols were harmonized, and treatment variations were analyzed as covariates.

### 2.4. Statistical Methods

Analyses were conducted using R (version 4.2.0; The R Foundation for Statistical Computing, Vienna, Austria) with descriptive statistics for demographic and clinical data. Continuous variables were presented as mean (standard deviation) or median (interquartile range [IQRs]). Categorical variables were presented as frequencies (%). Kolmogorov–Smirnov tests were used to assess data distributions. Missing data were managed using multiple imputations by chained equations with subsequent sensitivity analyses to determine imputation robustness and validate missing data assumptions.

Firth’s penalized logistic regression was used to identify severe disease risk factors while addressing small-sample bias. Predictors were selected based on clinical relevance and significance in univariate analysis (*p* < 0.20). Sensitivity analyses confirmed these findings, and odds ratios (ORs) with 95% confidence intervals (CIs) were calculated for balanced and unbalanced datasets. Adjustments for multiple comparisons were implemented using the Benjamini–Hochberg correction with a false discovery rate of 0.05. All significant associations persisted in the postadjustment analysis.

Validation showed reliable performance (Hosmer–Lemeshow test, *p* = 0.82; area under the curve, 0.83). Overfitting was reduced using LASSO, cross-validation, and the selection of strong predictors. Bootstrapping (1000 resamples) confirmed the effect stability, whereas sensitivity analyses and stratification validated the high ORs. A joint research team from the WHO and the Ministry of Health, comprising clinicians and epidemiologists, convened to review and validate the mpox data and to develop evidence-based control recommendations.

### 2.5. Ethics Approval

The WHO African Region Ethics Review Committee (AFR/ERC/2024/9.6) approved the generic WHO Mpox Transmission Investigation Protocol developed by the mpox technical team at the WHO headquarters in collaboration with partners. The Burundi National Ethics Committee (Décision CNE/33/2024 du Comité National d’Ethique) authorized this study with the use of a modified version of the protocol that ensures participant rights and data confidentiality through anonymization of data. The study adhered to the Strengthening the Reporting of Observational Studies in Epidemiology checklist. Images included in this paper were obtained with written informed consent for scientific publication. All identifying information was removed in compliance with institutional guidelines and data protection regulations.

## 3. Results

### 3.1. Participant Characteristics

Between 25 July 2024, and 26 October 2024, there were 850 laboratory-confirmed cases of mpox in Bujumbura, Burundi. The distribution of cases was as follows: 452 (53.1%) from Clinic Prince Louis Rwagasore, 349 (41.1%) from Centre Hospitalo-Universitaire de Kamenge, 46 (5.4%) from Hôpital Militaire de Kamenge, and 3 patients (0.4%) under home-based care. The affected population comprised 462 males (54.4%) and 388 females (45.6%), resulting in a male-to-female sex ratio of 1.2:1. Among females, 7 (1.8%) were pregnant. The median duration of hospital stay was 10 days (IQR: 7–14 days).

The median age was 20.3 years (IQR, 22.0; range, 3 months–71 years). Age distribution analysis showed that children aged < 5 years (13.6%, n = 115) and school-age children (5–15 years; 25.2%, n = 213), that is, pediatric cases (<16 years), constituted 38.8% (n = 328) of the total cases. Young adults (16–29 years old) represented 33.1% (n = 280) of the total patient population. Among the 522 patients aged > 16 years, individuals were single (50.6%, n = 264) or married (34.7%, n = 181), with an unknown status in 77 patients (14.7%).

Occupational data were available for 465 individuals, with the largest group comprising primary and secondary students (34.8%, n = 162), followed by merchants and traders (14.0%, n = 65). Health care professionals, military/police, and university students accounted for 0.9% (n = 4), 3.4% (n = 16), and 2.8% (n = 13) of the sample, respectively (Table 1).

### 3.2. Transmission Patterns

Of 850 laboratory-confirmed mpox cases in Bujumbura, Burundi, 70 secondary cases provided complete transmission pattern data. Among these 70 cases, two primary transmission routes were identified: sexual contact (58.6%, 41/70) and household exposure (38.6%, 27/70). Contact history analysis revealed exposures through sex workers (35.7%, 25/70), spouses (22.9%, 16/70), other household members (38.6%, 27/70), and healthcare personnel (2.8%, 2/70). Transmission events predominantly occurred in household settings (62.9%, 44/70). Local transmission was predominant, with 95.7% (67/70) of cases reporting no recent travel history.

### 3.3. Clinical Manifestations

**Figure 1:**
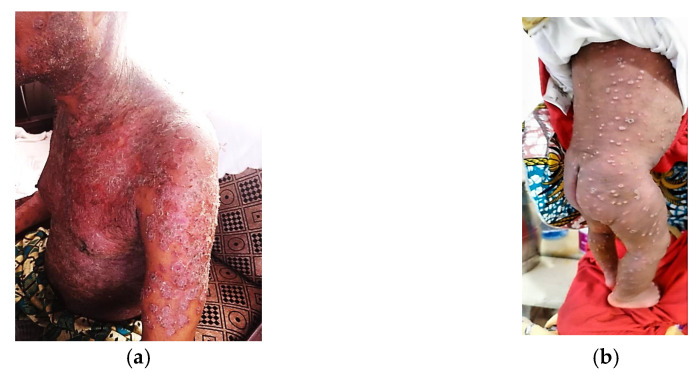
(**a**): Generalized mpox skin lesions with desquamation in immunocompromised patient, Burundi, 2024. (**b**): Generalized mpox skin lesions in 21-day-old infant, Burundi, 2024.

**Figure 2:**
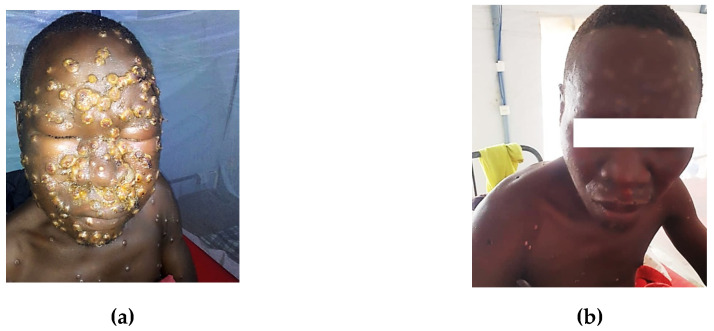
(**a**,**b**): Facial mpox lesions with conjunctivitis: before and after resolution, Burundi, 2024.

Clinical manifestations among the 835 laboratory-confirmed mpox cases with complete data demonstrated a characteristic pattern of presentation. Cutaneous and mucocutaneous manifestations were predominant, with generalized rash being the most frequent clinical feature (85.9%, n = 717) (Figure 1a,b and Figure 2a,b). Genital lesions were observed in nearly half of the cases (46.9%, n = 392), followed by oral lesions (27.5%, n = 230) and anorectal lesions (21.3%, n = 178). Constitutional symptoms were also common, with fever ≥38.5 °C present in more than half of the cases (54.5%, n = 455). Systemic involvement was evidenced by significant rates of asthenia/fatigue (35.1%, n = 293), local/regional adenopathy (33.3%, n = 278), and headache (31.6%, n = 264). Musculoskeletal symptoms were notable, with muscle pain and back pain affecting 21.7% (n = 181) and 17.5% (n = 146) of cases, respectively. Less frequent manifestations included respiratory symptoms (8.1%, n = 68) and gastrointestinal symptoms such as vomiting/nausea (2.0%, n = 17), suggesting a lower prevalence of non-cutaneous organ system involvement.

The age distribution analysis of mucocutaneous manifestations revealed distinct patterns across different age groups (Table 2).

**Figure 3:**
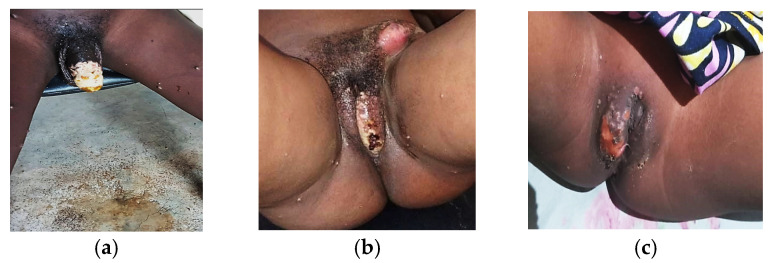
(**a**): Necrotic penile mpox lesion with purulent discharge in young adult male, Burundi, 2024. (**b)**: Genital pustular mpox lesions with adenopathy in adult female, Burundi, 2024. (**c**): Mpox genital lesion in 4-year-old female with no evidences of sexual transmission, Burundi, 2024.

Genital lesions demonstrated the highest prevalence in the 20–29 years age group (72.2%, 151/209) and the 30–39 years age group (65.4%, 100/153). In the pediatric population, genital manifestations were observed in 8.7% (10/115) of children aged 0–4 years, while 54.9% (39/71) of adolescents aged 16–19 years presented with genital lesions (Figure 3a–c).

Anorectal lesions exhibited an age-dependent distribution pattern, with the highest prevalence in the 50–59 years age group (100%, 9/9), followed by the 40–49 years age group (41.9%, 26/62). In the pediatric population, anorectal manifestations were documented in 11.3% (13/115) of children aged 0–4 years and 8.9% (19/213) of children aged 5–15 years.

Oral manifestations were observed across all age groups, with the highest prevalence in the 50–59 years age group (100%, 9/9) and the 40–49 years age group (61.3%, 38/62). The frequency of oral lesions in children aged 0–4 years was 20.9% (24/115), while in young adults aged 20–29 years and 30–39 years, the prevalence was 23.0% (48/209) and 37.3% (57/153), respectively. Multiple concurrent mucocutaneous manifestations were observed, particularly in the 50–59 years age group, where all cases presented with both anorectal and oral lesions.

### 3.4. Clinical Complications and Comorbidities

Complications occurred in 3.1% of the patients (n = 26) (Table 3). Secondary complications included vaginitis (1.1%, n = 9), genital ulceration (0.5%, n = 4), complicated pyelonephritis with vaginitis (0.1%, n = 1), and Fournier gangrene (0.1%, n = 1). Although lymphadenopathy is commonly observed as a characteristic clinical feature of mpox, it was analyzed as a presenting symptom rather than a complication.

Vaginitis was the most common complication at a rate of 1.1% overall (n = 9; 2.3% among 388 females; 9.1% among women aged ≥16 years). No deaths were reported in this study. Comorbidities were infrequent and included diabetes (0.35%, n = 3), renal insufficiency (0.24%, n = 2), hypertension (0.08%, n = 1), and malignancy (0.08%, n = 1).

A history of either prior or concurrent sexually transmitted infections was reported in 7.9% (n = 67) of patients. Most participants were HIV-negative (92.4%, n = 785), whereas 3.3% (n = 28) were HIV-positive, and 3.9% (n = 33) had an unknown status (not tested or data not available). Among those who tested positive for HIV, 46.4% (n = 13) were aware of their HIV-positive status, whereas 53.6% (n = 15) were newly diagnosed through testing during the clinical assessment for mpox. HIV-positive cases were mainly found among the age groups of 20–29 years and 30–39 years (n = 9 each), followed by 40–49 years (n = 5) and 16–19 years (n = 4) (Table 4). CD4 counts and HIV viral load data were not available for all HIV-positive cases.

### 3.5. Risk Factors for Mpox Complications

In the balanced dataset, Firth’s penalized multivariate logistic regression identified conjunctivitis as the most significant predictor of complications (OR, 27.30; 95% CI, 7.67–122.23; *p* < 0.001). Other notable predictors included pharyngitis (sore throat) (OR, 12.63; 95% CI, 5.78–30.21; *p* < 0.001) and genital edema (OR, 5.66; 95% CI, 1.55–23.28; *p* = 0.008). Conversely, generalized rash lesions (OR, 0.10; 95% CI, 0.04–0.24; *p* < 0.001) and oral lesions (OR, 0.20; 95% CI, 0.07–0.55; *p* = 0.001) were minimally associated with the occurrence of complications (Table 4). Low odds were associated with back pain (OR, 0.05; 95% CI, 0.01–0.43; *p* = 0.006), chills/sweats (OR, 0.03; 95% CI, 0.0002–0.30; *p* < 0.001), and local lymphadenopathy (OR, 0.24; 95% CI, 0.08–0.62; *p* = 0.003). These findings were also supported by the unbalanced dataset, particularly the strong associations with conjunctivitis (OR, 45.44; 95% CI, 5.98–464.05; *p* < 0.001) and vaginal lesions (OR, 8.88; 95% CI, 2.45–39.30; *p* < 0.001) (Table 5).

### 3.6. Disease Severity

Of the 850 laboratory-confirmed mpox cases followed prospectively, 566 (66.6%) remained mild throughout their clinical course (≤5 skin lesions), 180 (21.2%) progressed to moderate disease (6–100 lesions or mild complications), and 104 (12.2%) progressed to severe disease (≥100 lesions or serious complications such as secondary bacterial infections, encephalitis, pneumonia, genital necrosis, or ocular involvement requiring intensive clinical management).

## 4. Discussion

The results of our prospective cohort study of patients with laboratory-confirmed mpox who were hospitalized in Bujumbura, Burundi, highlight the necessity of a tailored, risk-based approach for treating patients with mpox. Our findings revealed critical clinical features of mpox, such as a high occurrence of generalized rash lesions (85.9%) and fever (54.5%) and slight male predominance (54.4%). The overall proportion of patients who experienced complications was lower (3.1%) than that in previous reports, and no deaths were noted during the study period.

Conjunctivitis and sore throat were significant predictors of complications, with ORs of 27.30 (95% CI, 7.67–122.23) and 12.63 (95% CI, 5.78–30.21), respectively. We observed an inverse association between local lymphadenopathy, widespread rash, and oral lesions. Our data imply that a skin-dominated disease could result in a favorable prognosis, likely because of its early recognition and rapid treatment. Additionally, some systemic signs, such as conjunctivitis and sore throat, were important indicators of severe disease status, necessitating continuous monitoring of the affected individuals.

Unlike the male predominance (96.4%) in the global outbreak, particularly among men who have sex with men [12], our African cohort demonstrated balanced transmission patterns (54.4% males) [13,14]. The prevalence of fever (54.5%) and widespread rash (85.9%) in a study by Yon et al. [15] was similar to that in our cohort and comparable to both global data for clade II MPXV (62.3% and 85.7%, respectively) and historical clade I MPXV data from DRC-endemic regions (fever, 72.4%; rash, 89.3%) [WHO. Multi-country outbreak of mpox, External situation report#31—22 December 2023]. These clinical variations, observed despite shared clade Ib circulation with DRC, suggest evolving transmission patterns and necessitate further investigation of regional epidemiological differences.

In this analysis, conjunctivitis and sore throat were significant predictors of complications, indicating a substantial prognostic value. The correlation between conjunctivitis and subsequent disease severity aligns with the findings of a previous study by García et al., 2024 [16], who identified ocular symptoms as indicators of severity. While conjunctivitis can be an indicator of mpox severity, it is not the sole predictor. The overall severity of mpox is influenced by various factors, including the patient’s immune status and the specific viral clade involved.

These findings highlight the need for vigilant assessment and monitoring of conjunctivitis and/or sore throat early in the course of the illness. Our findings regarding the inverse associations of specific symptoms are in line with the broad understanding that early detection and treatment play a crucial role in minimizing the disease burden [17].

These mucosal manifestations (conjunctivitis and sore throat) may indicate enhanced viral tropism in ocular and mucosal tissues, suggesting an increase in both the viral burden and the systemic inflammatory response. The predictive value of sore throat for the development of severe disease supports the observations of De la Herrán-Arita et al. [18]; however, our study extends this significance, indicating a stronger association than previously documented.

Similarly, the negative correlation of local lymphadenopathy contrasts with the findings of Álvarez-Moreno et al. [19], which showed neutral associations. This discrepancy may stem from differences in immune responses, suggesting that localized lymphadenopathy may reflect efficient viral containment. The age-related vulnerability observed in our study aligns with the findings of Cho et al. regarding the severity of mpox in older populations [20].

The strong association of conjunctivitis with severe disease emphasizes the potential significance of noncutaneous manifestations as indicators of systemic progression [16,21]. Analyses of the time to treatment demonstrated that the protective associations persisted even after adjusting for delays in seeking care, thus strengthening the hypothesis of a biological mechanism. Variance inflation factors and correlation analyses validated the independence of the predictors, and stepwise variable selection mitigated potential issues of collinearity. Evidence suggests that HIV infection without immune suppression is not a direct risk factor but instead correlates through shared behavioral and immunological factors [18].

The presence of skin lesions without concurrent mucosal or systemic symptoms was associated with few complications, supporting the observation of Ogoina et al. that isolated cutaneous involvement may not indicate a severe disease course [21].

Our findings from Burundi demonstrate distinctive patterns in clinical manifestations compared with previously reported data from African settings. Cutaneous manifestations, particularly generalized rash, constituted the predominant clinical feature, affecting 85.9% (717/835) of cases, aligning with the characteristic presentation patterns described by Cices et al. (2023) [22]. This high prevalence of cutaneous manifestations is consistent with established clinical patterns of mpox infection.

The therapeutic management of mpox infections remains challenging, particularly in resource-limited settings. While tecovirimat has been validated under an Expanded Access-Investigational New Drug protocol for mpox treatment, a study conducted in the DRC demonstrated that this antiviral did not improve clade I mpox lesion resolution time, despite its safety profile [23]. These findings support the current approach in resource-limited settings like Burundi, where emphasis on supportive care and complication management proves more practical than implementing complex antiviral protocols. [23]. Burundi health authorities have not requested Tecovirimat to date.

Monkeypox virus proteins govern host entry and clinical manifestations, with Apolipoprotein B mRNA-editing enzyme catalytic polypeptide-like 3 (APOBEC3) mutations affecting viral evolution in sub-clades Ib and IIb [24]. Clade I exhibits higher virulence than clade II [25], with mutation patterns in DRC impacting disease presentation [26]. These molecular modifications appear to influence both transmission dynamics and clinical presentation, with implications for cross-border surveillance and regional public health responses in Central Africa.

Recent genomic analyses reveal distinct characteristics across MPXV clades. Clade Ia exhibits limited human-to-human transmission with predominantly zoonotic patterns and higher mortality, particularly in young children. Conversely, clades Ib and IIb demonstrate enhanced human-to-human transmission with increased viral shedding but milder clinical manifestations [27]. Molecular characterization confirms circulation of clade Ib strains in Burundi with genetic similarity to South Kivu Province, DRC isolates [28].

Our analysis of 850 mpox cases in Bujumbura demonstrated that sexual contact was the predominant transmission route (58.6%), with a significantly higher proportion of household transmission (38.6%) compared to Western cohorts (5–10%) [29,30,31], reflecting distinct regional variations in social structures. The distribution of genital lesions (46.9% overall) exhibited clear age-specific patterns, with higher prevalence in ages 20–29 (72.2%) and 30–39 (65.4%) years compared to children aged 0–4 (8.7%) and 5–15 (23.0%) years. Since mpox is a mucocutaneous infection capable of affecting any skin or mucous membrane through direct contact, the presence of genital lesions—particularly in pediatric cases—does not necessarily indicate sexual transmission, as lesions can develop at any site of viral contact regardless of transmission route. While these patterns share similarities with HSV presentation, the predominantly syndromic approach to STI diagnosis in Burundi limits definitive differentiation between causes, especially considering that mpox and HSV can occur concurrently. These findings emphasize the importance of context-specific interventions addressing both sexual and non-sexual transmission routes [32,33,34,35,36], challenging the predominant global focus on sexual transmission as the primary route.

### 4.1. Study Limitations

Several methodological constraints must be considered when interpreting our findings. It was challenging to establish a temporal relationship between initial symptoms and disease progression, as patients presented with varying stages of illness (median time from symptom onset to hospitalization, 5.2 days; IQR, 3–8 days). Although our study provides valuable insights from multiple centers in Burundi, the findings should be interpreted within the context of our setting’s specific health care infrastructure and patient population. The wide CIs observed for conjunctivitis as a prognostic indicator suggest that this clinical feature should be evaluated along with other presenting symptoms and patient characteristics. Further assessment of this potential early warning sign in different clinical contexts would enhance our understanding of its prognostic value while maintaining vigilance for severe disease progression. Variations in treatment protocols across study centers may have influenced our risk factor analysis. In contrast, the limited duration of the study precluded the assessment of seasonal patterns in disease presentation and transmission dynamics.

Burundi’s mandatory hospitalization policy for laboratory-confirmed mpox cases has both strengths and limitations. Although this approach enabled comprehensive clinical documentation, it has potentially introduced selection bias by deterring individuals from seeking diagnoses. Although we achieved high retention rates, with 99.6% of patients completing their hospital stay, facilitated by the provision of free health care and nutritional support, this differs notably from settings such as the DRC, where socioeconomic constraints frequently lead to premature discharge.

Our E-value analysis (E = 3.2) indicated that substantial unmeasured confounding is necessary to nullify the observed associations. However, the dynamic nature of mpox manifestations complicates our analysis. Despite implementing standardized daily assessment forms, capturing the complex evolution of skin lesions and their relationship with systemic complications requires sophisticated temporal analysis. Future prospective studies incorporating detailed temporal data collection methods across diverse health care settings are required.

### 4.2. Clinical Implications

These findings highlight the need to prioritize monitoring conjunctivitis and sore throat as possible key predictors of severe mpox and to stratify risk by age and sex, particularly for older adults and women. The early detection of cutaneous symptoms and risk-based care pathways can optimize outcomes in resource-limited settings. The inverse association between local lymphadenopathy and complications suggests that it may represent a typical immune response rather than a marker of disease severity. Future studies should explore the immunological significance of lymphadenopathy patterns in mpox disease across different geographic regions. Developing standardized and locally adapted risk tools and investigating host and viral factors may enable personalized care. Research on immunocompromised populations, particularly those with advanced HIV, is essential to refine targeted treatments and understand severe mpox outcomes. With two-thirds of mpox cases meeting the criteria for mild disease in Burundi, in the absence of warning signs such as those found here, implementing structured home-based care strategies could optimize health care resources while maintaining effective disease surveillance and control.

## 5. Conclusions

This study provides a comprehensive analysis of mpox clinical manifestations and associated risk factors in Burundi and offers novel insights into the clinical progression of the disease. Conjunctivitis and sore throat are key predictors of severe disease, with conjunctivitis showing an exceptionally high prognostic value. These findings highlight the importance of mucosal symptoms and findings as red flags in clinical assessment. In contrast, generalized rash and lymphadenopathy limited to the local distribution in the body were associated with low severity. They may signify robust immune responses rather than signs of an advanced disease stage. Older age was identified as a risk factor, emphasizing the need for targeted monitoring and intervention in older patients who are at an increased risk of severe outcomes.

These findings have important clinical implications, especially in resource-limited settings, where the recognition of easily observable markers, such as conjunctivitis, sore throat, and skin lesions, can guide risk stratification and optimize patient management. This study provides a foundation for the development of evidence-based management protocols that emphasize risk-stratified care pathways and structured home-based strategies.

In conclusion, the inclusion of clinical markers in patient assessment protocols may aid the distribution of resources, enhancement of outcomes, and development of public health initiatives, particularly in endemic and resource-constrained areas. These results indicate a significant advancement in the global improvement of mpox management caused by clade Ib MPXV.

## Figures and Tables

**Table 1:** Reported occupations of patients with laboratory-confirmed mpox, Bujumbura, Burundi, July–October 2024.

Occupation	Count (*n* = 465 *)	Percent (%)
Primary and secondary students	162	34.8
Merchants and sellers	65	14.0
Commercial sex workers	22	4.7
Drivers	19	4.1
Military and police	16	3.4
Health care professionals	4	0.9
University students	13	2.8
Mechanics	11	2.4
Other	153	32.9

* Among those for whom data is available.

**Table 2:** Age distribution of mucocutaneous manifestations among laboratory-confirmed mpox cases, Burundi, 2023–2024.

Age Group (Years)	Total Cases n (%) ^1^	Genital Lesions ^2^ n (%) ^3^	Anorectal Lesions n(%) ^3^	Oral Lesions n (%) ^3^
0–4	115 (13.8)	10 (8.7)	13 (11.3)	24 (20.9)
5–15	213 (25.5)	49 (23.0)	19 (8.9)	41 (19.2)
16–19	71 (8.5)	39 (54.9)	14 (19.7)	12 (16.9)
20–29	209 (25.0)	151 (72.2)	45 (21.5)	48 (23.0)
30–39	153 (18.3)	100 (65.4)	51 (33.3)	57 (37.3)
40–49	62 (7.4)	38 (61.3)	26 (41.9)	38 (61.3)
50–59	9 (1.1)	5 (55.6)	9 (100.0)	9 (100.0)
≥60	3 (0.4)	0 (0.0)	1 (33.3)	0 (0.0)
Total	835 (100.0)	392 (46.9)	178 (21.3)	230 (27.5)

Note: ^1^ Percentages in the Total Cases column are calculated vertically (proportion of all cases). ^2^ Genital lesions include both penile and vulvovaginal manifestations. ^3^ Percentages in the lesion columns are calculated horizontally (proportion of age group total). Patients may present with multiple types of lesions simultaneously.

**Table 3:** Complications recorded among patients with laboratory-confirmed mpox, Bujumbura, Burundi, July–October 2024.

Primary Complication	Count	Percent (%)
No complications	820	96.5
Any complication	26	3.1
Vaginitis	9	1.1
Ulceration in the genital area	4	0.5
Conjunctivitis	2	0.2
Necrotic lesions in the scrotum/penis	2	0.2
Other specific complications (e.g., cellulitis, human immunodeficiency virus-related ocular lesions, genital necrosis, complicated pyelonephritis with vaginitis, Fournier gangrene)	≤1 each	<0.2 each
Secondary complication		
No secondary complications	807	94.9
Any secondary complication	43	5.1
Specific cases (e.g., complicated pyelonephritis, Fournier gangrene)	≤1 each	<0.2 each

**Table 4:** Demographic and clinical characteristics and comorbidities among patients with laboratory-confirmed mpox, Bujumbura, Burundi, July–October 2024.

Category	Subcategory	Count	Percent (%)
Comorbidity	STI history (No)	710	58.1
	STI history (Yes)	67	5.49
	Hypertension (No)	871	68.6
	Hypertension (Yes)	1	0.08
	Diabetes (No)	814	66.7
	Diabetes (Yes)	3	0.25
	Cancer (No)	844	69.1
	Cancer (Yes)	1	0.08
	Kidney failure (No)	843	69.0
	Kidney failure (Yes)	2	0.16
	Others (No)	204	16.67
	Others (Yes)	2	0.16
	Erythematous-squamous dermatosis	1	0.08
	Pyelonephritis complicated by vaginitis	1	0.08
HIV status	Negative	785	92.4
	Unknown	33	3.9
	Positive	28	3.3
Age (HIV-positive cases)	16–19 years	4	
	20–29 years	9	
	30–39 years	9	
	40–49 years	5	
	50+ years	1	

Stratified analyses across age and HIV status with interaction testing confirmed consistent associations despite varying effect magnitudes in the key subgroups. HIV, human immunodeficiency virus; STI, sexually transmitted infection.

**Table 5:** Risk factors associated with mpox complications: Firth’s penalized multivariate logistic regression analysis of laboratory-confirmed cases in Bujumbura, Burundi, July–October 2024.

Variables	Balanced Dataset		Unbalanced Dataset	
	OR (95% CI)	*p* Value	OR (95% CI)	*p* Value
Sore throat	12.63 (5.78–30.21)	<0.001 *	4.32 (1.15–19.61)	0.030
Conjunctivitis	27.30 (7.67–122.23)	<0.001 *	45.44 (5.98–464.05)	<0.001 *
Asthenia/fatigue	0.33 (0.10–0.93)	0.036	1.81 (0.35–9.58)	0.477
Muscle pain	2.86 (1.07–8.00)	0.037	1.06 (0.23–4.73)	0.940
Back pain	0.05 (0.01–0.43)	0.006 *	0.21 (0.01–2.01)	0.185
Chills/sweats	0.03 (0.00–0.30)	<0.001 *	0.27 (0.00–3.23)	0.344
Local lymphadenopathy	0.24 (0.08–0.62)	0.003 *	0.13 (0.01–1.06)	0.058
Genital edema	5.66 (1.55–23.28)	0.008 *	3.67 (0.58–22.69)	0.162
Generalized lesions	0.10 (0.04–0.24)	<0.001 *	0.36 (0.05–1.69)	0.203
Oral lesions	0.20 (0.07–0.55)	0.001 *	0.44 (0.07–2.01)	0.304
Vaginal lesions	3.44 (1.68–7.14)	<0.001 *	8.88 (2.45–39.30)	<0.001 *

OR, odds ratio; CI, confidence interval. * Statistically significant after Benjamini–Hochberg correction (false discovery rate = 0.05). Only the variables with significant associations in at least one dataset are shown. Model fit: Hosmer–Lemeshow test *p* = 0.82; area under the curve = 0.83.

## Data Availability

All relevant data generated during this study are included in the manuscript.

## Abbreviations

The following abbreviations are used in this manuscript:

AIDS	Acquired Immunodeficiency Syndrome
CD4	Cluster of Differentiation 4
CI	Confidence Interval
DNA	Deoxyribonucleic Acid
DRC	Democratic Republic of the Congo
E E-value	(statistical measure)
HIV	Human Immunodeficiency Virus
IQR	Interquartile Range
LASSO	Least Absolute Shrinkage and Selection Operator
MPXV	Orthopoxvirus monkeypox
OR	Odds Ratio
*p*	Probability Value
PCR	Polymerase Chain Reaction
RNA	Ribonucleic Acid
SD	Standard Deviation
STI	Sexually Transmitted Infection
STROBE	Strengthening the Reporting of Observational Studies in Epidemiology
U.S.	United States
UCL	University College London
USAID	United States Agency for International Development
WHO	World Health Organization
